# 5-Ethyl-3,7-dioxa-1-azabicyclo[3.3.0]octan (EDAO): MAK-Begründung, Nachtrag

**DOI:** 10.34865/mb774735kskd10_4ad

**Published:** 2025-12-22

**Authors:** Andrea Hartwig, MAK Commission

**Affiliations:** 1 Institut für Angewandte Biowissenschaften. Abteilung Lebensmittelchemie und Toxikologie. Karlsruher Institut für Technologie (KIT) Adenauerring 20a, Geb. 50.41 76131 Karlsruhe Deutschland; 2 Ständige Senatskommission zur Prüfung gesundheitsschädlicher Arbeitsstoffe. Deutsche Forschungsgemeinschaft, Kennedyallee 40, 53175 Bonn, Deutschland. Weitere Informationen: Ständige Senatskommission zur Prüfung gesundheitsschädlicher Arbeitsstoffe | DFG

**Keywords:** 5-Ethyl-3,7-dioxa-1-azabicyclo[3.3.0]octan, Nase, Reizwirkung, Formaldehydabspalter, Formaldehyd-Abspalter, MAK-Wert, maximale Arbeitsplatzkonzentration, Kanzerogenität, Keimzellmutagenität, Entwicklungstoxizität, Sensibilisierung

## Abstract

The German Senate Commission for the Investigation of Health Hazards of Chemical Compounds in the Work Area (MAK Commission) summarized and re-evaluated the data for 5-ethyl-3,7-dioxa-1-azabicyclo[3.3.0]octane (EDAO) [7747-35-5] to derive an occupational exposure limit value (maximum concentration at the workplace, MAK value) considering all toxicological end points. Relevant studies were identified from a literature search and also unpublished study reports were used. The substance is irritating to the skin and corrosive to the eyes of rabbits. EDAO is not expected to occur in aerosol form because of its vapour pressure. EDAO releases formaldehyde in aqueous solution. For this reason, the effects are attributed to the hydrolysis products formaldehyde and 2-amino-2-ethyl-1,3-propanediol. It is assumed that EDAO hydrolyses to formaldehyde in the respiratory tract. In the worst case, two formaldehyde molecules per molecule of EDAO could be released in the respiratory tract. Taking this into consideration, a MAK value of 0.15 ml/m^3^ has been derived for EDAO in analogy to the MAK value of 0.3 ml/m^3^ for formaldehyde. There are no studies that investigated the carcinogenicity, toxicity or genotoxic potential of EDAO in the upper respiratory tract or nose, the likely target organs. The substance has genotoxic potential in vitro, presumably due to the release of formaldehyde. Formaldehyde has been classified in Carcinogen Category 4 because it causes nasal tumours at concentrations that exceed the detoxification capacity of that tissue. Thus, in analogy to the classifications for formaldehyde, the substance has been assigned to Carcinogen Category 4 and Germ Cell Mutagenicity Category 5. In rats, the NOAELs for perinatal toxicity and teratogenicity of EDAO are 150 mg/kg and 250 mg/kg body weight, respectively. As the margins between the NOAELs and the MAK value are sufficiently large, EDAO has been assigned to Pregnancy Risk Group C. EDAO has skin sensitizing potential. Therefore, the “Sh” designation has been retained. There are no data for respiratory sensitization. Skin contact is not expected to contribute significantly to systemic toxicity.

**Table:** 

**MAK-Wert (2023)**	**0,15 ml/m^3^ (ppm) ≙ 0,89 mg/m^3^**
**Spitzenbegrenzung (2023)**	**Kategorie I, Überschreitungsfaktor 2**
	
**Hautresorption**	**–**
**Sensibilisierende Wirkung (2004)**	**Sh**
**Krebserzeugende Wirkung (2023)**	**Kategorie 4**
**Fruchtschädigende Wirkung (2023)**	**Gruppe C**
**Keimzellmutagene Wirkung (2023)**	**Kategorie 5**
	
**BAT-Wert**	**–**
	
Synonyma	1-Aza-3,7-dioxa-5-ethylbicyclo[3.3.0]octan 7-Ethylbicyclooxazolidin 7a-Ethyldihydro-1H,3H,5H-oxazolo[3,4-c]oxazol Oxazolidine-E
Chemische Bezeichnung (IUPAC-Name)	7*a*-Ethyl-1,3,5,7-tetrahydro-[1,3]oxazolo[3,4-c][1,3]oxazol
CAS-Nr.	7747-35-5
Molmasse	143,18 g/mol
Reinheit	98,55 % (ECHA 2013) 96,85 % (Case Consulting Laboratories 2002)
Schmelzpunkt	1 °C (ECHA 2013)
Siedepunkt bei 20 hPa	187,0 °C (ECHA 2013)
Dichte bei 20 °C	1,08 g/cm^3^ (Case Consulting Laboratories 2002; ECHA 2013; Greim 1993)
Dampfdruck bei 25 °C	0,606 hPa (ECHA 2013); 1,04 hPa (ber., US EPA 2022)
bei 20 °C	0,376 hPa (ECHA 2013)
log K_OW_	–1,1 (pH 5); –0,32 (pH 7); –0,31 (pH 9) (ECHA 2013)
Löslichkeit	mischbar mit Wasser (ECHA 2013), Löslichkeit in Hexan, Chloroform, Ethylacetat, Methanol > 100 g/l (Kurume Research Laboratories 1993), zudem löslich in Ethanol, Aceton, Benzol, chlorierten Kohlenwasserstoffen (Greim 1993)
pH-Wert	8,86 (96%ig) (The Dow Chemical Company 2007 a) 10,46 (0,1 M) (The Dow Chemical Company 2005) 10 (1%ig) (ECHA 2013)
pKs-Wert	4,0 (ECHA 2013)
**1 ml/m^3^ (ppm) ≙ 5,9 mg/m^3^**	**1 mg/m^3^ ≙ 0,17 ml/m^3^ (ppm)**
	
Hydrolysestabilität	hydrolysiert unter Abspaltung von Formaldehyd und 2-Amino-2-ethyl-1,3-propandiol, Hydrolysegeschwindigkeit abhängig von pH-Wert und Temperatur (Abschnitt 3.2)
Verwendung	Ledergerbung (ECHA 2013); als Biozid in Schneidflüssigkeiten, Schneidölen, Schmiermitteln und Additiven; Lederbearbeitung, Gerben; UV-Filter, Konservierungsmittel und antimikrobielles Mittel in kosmetischen Produkten, in Haushaltsreinigungsprodukten (Geschirrspül- und Waschflüssigkeiten, Oberflächenreiniger und -polituren, in Farben und Beschichtungen, in Klebstoffen; als Korrosionsinhibitor; als Urethanschaumkatalysator (NICNAS 2020)
Einsatzverbote	in Mundhygienemitteln und Mitteln, die auf Schleimhäute aufgetragen werden (Europäisches Parlament und Europäischer Rat 2022)
Einsatzkonzentration	3–5 % in Mitteln zur Ledergerbung (Serfass 2024), bis zu 0,3 % in Kosmetika (Europäisches Parlament und Europäischer Rat 2022), Einsatzkonzentration als Biozid um 0,1 % (Greim 1993)

Hinweis: Formaldehydabspalter

Es liegen eine Begründung (Greim 1993) und Nachträge zur sensibilisierenden Wirkung (Greim 1996, 2004) vor.

Dieser Nachtrag wird erstellt, da eine weitere Hydrolyse-Studie zur Verfügung steht. Zudem erfolgt eine Neubewertung der Daten zur Formaldehydabspaltung nach aktuellem Vorgehen der Kommission (DFG 2024).

Bei Kontakt mit Wasser hydrolysiert 5-Ethyl-3,7-dioxa-1-azabicyclo[3.3.0]octan (EDAO) zu zwei Molekülen Formaldehyd und einem Molekül 2-Amino-2-ethyl-1,3-propandiol.

## 1 Allgemeiner Wirkungscharakter

EDAO ist ein Formaldehydabspalter. Die Hydrolyse erfolgt bei 25 °C mit einer Halbwertszeit von 1,9 Stunden im neutralen und schneller im sauren Bereich. Formaldehyd wirkt bei inhalativer Exposition kanzerogen an der Nase von Ratten.

Am Auge wirkt EDAO ätzend und an der intakten Haut reizend. Es liegen positive Befunde zur kontaktallergenen Wirkung von EDAO beim Menschen vor. Zwei tierexperimentelle Untersuchungen am Meerschweinchen weisen auf ein hautsensibilisierendes Potenzial hin. Zur atemwegssensibilisierenden Wirkung liegen keine Daten vor.

Nach 28-tägiger Schlundsondengabe führt EDAO ab einer Konzentration von 21,7 mg/kg KG bei männlichen Sprague-Dawley-Ratten zu Reizungen der Magenschleimhaut, die sich nach 90-tägiger Gabe zu Entzündungen verstärken.

In vitro ist EDAO negativ in bakteriellen Mutagenitätstests, Chromosomenaberrationstests und zeigt keine verstärkte DNA-Reparatursynthese (UDS). Eine positive Reaktion im TK^+/–^-Test ist auf die Freisetzung von Formaldehyd zurückzuführen und weist aufgrund der deutlichen Zunahme kleiner Kolonien auf eine Induktion von Chromosomenaberrationen hin. In In-vivo-Tests wird keine systemische Genotoxizität an Nagern beobachtet.

In einer 2-Generationen-Studie zeigen sich nach oraler Gabe an Ratten keine Effekte auf die Fertilität. Bei Ratten treten nach oraler Gabe vom 6. bis zum 15. Gestationstag bei 650 mg EDAO/kg KG und Tag Ossifikationsverzögerungen, Fehlbildungen und Spätresorptionen bei maternaltoxischen Dosierungen auf. Die Entwicklungstoxizität ist charakterisiert durch ausgeprägte Unterschiede zwischen einzelnen Würfen.

## 2 Wirkungsmechanismus

EDAO hydrolysiert unter Abspaltung von Formaldehyd. Bei einer 1%igen EDAO-Lösung beträgt der pH-Wert 10. EDAO und die Hydrolyseprodukte sind für die reizende Wirkung an Haut, Auge und Schleimhäuten verantwortlich (ECHA 2013). Es ist wahrscheinlich, dass die pH-Werte, die bei hohen Verdünnungen erzielt werden, eher den pH-Wert des Hydrolyseproduktes 2-Amino-2-ethyl-1,3-propandiol als den pH-Wert von EDAO widerspiegeln (Serfass 2024).

Das Hydrolyseprodukt 2-Amino-2-ethyl-1,3-propandiol ist mit pH 10,8 (0,1 M in Wasser) stark basisch und zeigt ebenfalls eine reizende Wirkung an der Haut und eine ätzende Wirkung am Auge (Greim 1995; IFA 2023 a).

Formaldehyd wirkt kanzerogen an der Nase (Greim 2000).

EDAO sowie Formaldehyd waren mutagen in einem TK^+/–^-Test mit L5178Y-Mauslymphomzellen. Nach Zugabe von Formaldehyd-Dehydrogenase traten jedoch weder mit EDAO noch mit Formaldehyd erhöhte Mutationen auf. Daraus kann geschlossen werden, dass durch die hydrolytische Abspaltung entstehendes Formaldehyd den genotoxischen In-vitro-Effekt bewirkte (Charles et al. 2005).

## 3 Toxikokinetik und Metabolismus

Nach OECD-Prüfrichtlinie 111 wurde die Hydrolyse des EDAOs bei verschiedenen pH-Werten bei 15 °C und 25 °C bestimmt. Bei pH 7 und 25 °C wurde die Hydrolysebestimmung wiederholt. Die Hydrolyserate nahm bei einer Konzentration von 20 mg EDAO/l (Reinheit 96,85 %) mit steigendem pH-Wert der Lösung ab. Bei pH 7 betrug die mittlere Halbwertszeit 1,9 Stunden bei einer Temperatur von 25 °C (siehe Tabelle 1; The Dow Chemical Company 2003).

**Tab. 1:** Halbwertszeiten der Hydrolyse von 0,002 % EDAO bei verschiedenen pH-Werten und Temperaturen (The Dow Chemical Company 2003)

**Temperatur [°C]**	**Halbwertszeit [h]**		
	**pH 4**	**pH 7**	**pH 9**
15	0,09	7,9	9,6
25	0,1	2,2	3,8
25		1,6	

Zur Identifikation der Hydrolyseprodukte wurde 160 mg EDAO/l zu einer auf pH 4 gepufferten Lösung gegeben. Die Konzentration des EDAOs nahm innerhalb von 0,5 Stunden von 160 auf 60 mg/l ab und blieb dann für die folgenden drei Stunden konstant. Versuche, die Abbauprodukte durch HPLC-Massenspektrometrie zu identifizieren, waren erfolglos (The Dow Chemical Company 2003).

Basierend auf diesen Daten wurde die Halbwertszeit unter den pH- und Temperaturbedingungen des TK^+/–^-Tests (pH 7–7,4 und 37 °C) auf eine Stunde geschätzt (The Dow Chemical Company 2003).

### 3.1 Aufnahme, Verteilung, Ausscheidung

In einer Schlundsonden-Studie nach OECD-Prüfrichtlinie 417 wurden einmalig 0, 5 oder 200 mg ^14^C-EDAO/kg KG (7a-Ethyl-1,7-dihydro[1,7-^14^C]oxazolo[3,4-c]oxazol, d. h. die Markierung war nicht an den Formaldehyd-C-Atomen, 25 µCi/kg) in Maiskeimöl an je vier männliche und weibliche Crl:CD(SD)-Ratten gegeben. Die Bestimmung der Radioaktivität erfolgte zu verschiedenen Zeitpunkten. Die Daten zur Resorption, Verteilung und Ausscheidung sind in der Tabelle 2 dargestellt. In der Haut fanden sich 3–4 % der Radioaktivität wieder. Dies war nach Meinung der Autoren unerwartet viel. Eine weitere Gruppe von je vier männlichen und weiblichen Crl:CD(SD)-Ratten erhielt 14 Tage lang täglich nicht markiertes EDAO und am 15. Tag 5 mg ^14^C-EDAO/kg KG. Die fünfzehntägige EDAO-Gabe führte weder zu einer kürzeren oder längeren Verweildauer im Körper noch zu einem veränderten Verhältnis von Radioaktivität in Urin und Faeces und damit nicht zu einer Induktion des eigenen Metabolismus. Die Elimination der Radioaktivität aus dem Plasma erfolgte zweiphasig, wobei der größte Teil der Elimination während der schnellen Eliminationsphase (Halbwertszeit: 0,1–0,5 Stunden) stattfand, gefolgt von einer langsameren Eliminationsphase (Halbwertszeit: 4–5 Stunden). Die an rote Blutzellen gebundene Radioaktivität war 20–50 % niedriger als die im Plasma. Die Eliminationshalbwertzeit der an rote Blutzellen gebundenen Radioaktivität war 2,6–3,0-fach länger als im Plasma. Der Großteil des zugeführten EDAOs wurde mit dem Urin ausgeschieden (83–97 %) und ein kleinerer Teil von 9–15 % mit den Faeces. Es gab anhand der biliär ausgeschiedenen Radioaktivität ab der niedrigsten Dosis Hinweise auf einen enterohepatischen Kreislauf zwischen zwei und sechs Stunden nach der Applikation; dies wurde bei den Tieren der hohen Dosis deutlicher. Es fand eine fast vollständige Hydrolyse des EDAO zu 2-Amino-2-ethyl-1,3-propandiol und Formaldehyd statt. Die mittlere Gesamtwiederfindung der Radioaktivität lag zwischen 95 und 109 % (bei 34 von 40 Tieren zwischen 94 und 101 %) der verabreichten Dosis (The Dow Chemical Company 2008 a). Die orale Resorption kann als vollständig angenommen werden, da der mit den Faeces ausgeschiedene Teil sehr wahrscheinlich auch resorbiert worden ist, weil auch nach dermaler Applikation 4 % der Dosis in den Faeces gefunden wurden.

**Tab. 2:** Aufnahme, Ausscheidung und Verteilung von ^14^C-EDAO nach oraler Gabe an Ratten (The Dow Chemical Company 2008 a)

**Dosierung**	**5 mg ^14^C-EDAO/kg KG** **einmalig**		**200 mg ^14^C-EDAO/kg KG** **einmalig**		**5 mg EDAO/kg KG, 14 Tage;** **15. Tag 5 mg ^14^C-EDAO/kg KG ** **einmalig**	
	**♂**	**♀**	**♂**	**♀**	**♂**	**♀**
Resorption, gesamt (Urin, Faeces, Gewebe)	93 %	100 %	99 %	98 %	100 %	100 %
Radioaktivität AUC im Plasma	5,5 µg h/g	6,4 µg h/g	270 µg h/g	281 µg h/g	–	–
Gehalt 15 min nach der Gabe						
Ganzkörper	91–94 %		–	–	–	–
Magen-Darm-Trakt	69 %					
Leber	2 %					
Nieren	0,5–1,3 %					
Haut	3–4 %					
Gehalt 6 h nach der Gabe						
Ganzkörper	40 %					
Magen-Darm-Trakt	15 %					
Magen	1–2 %					
Leber	3–5 %					
Nieren	ca. 1 %					
Zeit bis 1 % der Dosis im Körper erreicht	nach 168 h		nach 144 h		nach 168 h	
Ausscheidung Urin						
innerhalb von 6 h	43 %	43 %	–	–	–	–
innerhalb von 12 h	64 %	70 %	66 %	61 %	75 %	75 %
12–24 h nach der Gabe	9 %	11 %	11 %	12 %	9 %	11 %
bis 168 h p. a.	83 %	90 %	87 %	82 %	93 %	97 %
Ausscheidung Faeces	9 %	9 %	11 %	15 %	11 %	13 %
Gewebe	1 %	1 %	1 %	1 %	1 %	1 %
Wiederfindung	95 %	101 %	100 %	100 %	109 %	108 %
HWZ erste Phase	0,1–0,5 h		0,1–0,2 h		–	–
HWZ zweite Phase	4–5 h		ca. 5 h		–	–
Plasma C_max_	2,1 µg/g (t_max_ 0,25 h)	1,87 µg/g (t_max_ 0,25 h)	60,2 µg/g (t_max_ 0,25 h)	44,7 µg/g (t_max_ 0,25 h)	–	–

AUC: „area under the curve“; C_max_: maximale Konzentration; h: Stunde; HWZ: Halbwertszeit; min: Minuten; p. a.: nach der Gabe; t_max_: Zeitpunkt der maximalen Konzentration

Nach OECD-Prüfrichtlinie 427 wurden je vier männliche und weibliche Crl:CD(SD)-Ratten sechs Stunden lang dermal, semiokklusiv gegen 5 mg ^14^C-EDAO/kg KG (7a-Ethyl-1,7-dihydro[1,7-^14^C]oxazolo[3,4-c]oxazol, 20,92 mCi/mmol, Reinheit 99,1 %) in Polyethylenglykol (PEG 400) exponiert. Es wurde eine Lösung von 8,75 mg/g hergestellt und 120 µl auf 10 cm^2^ appliziert. Dies entspricht einer Dosis von 1 mg/10 cm^2^. Die dermale Resorption bei männlichen bzw. weiblichen Tieren betrug 27 ± 3 % und 25 ± 5 %. Das heißt, es wurden maximal 0,27 mg während der sechsstündigen Applikation resorbiert. Das entspricht auf eine Stunde berechnet 0,27 mg/6 h/10 cm^2^ = 4,5 µg/cm^2^. Die Wiederfindung der Radioaktivität betrug nur 66–70 %. Im gesamten Sammelzeitraum (168 Stunden) wurden 19 % bzw. 15 % der verabreichten Dosis im Urin von männlichen und weiblichen Ratten wiedergefunden, wobei nur 9–11 % der verabreichten Dosis innerhalb der ersten 24 Stunden ausgeschieden wurden. Das entspricht 46–65 % der gesamten ausgeschiedenen Radioaktivität. Die vollständige Urinausscheidung machte im Verlauf der Studie 64–80 % der resorbierten Dosis aus. Mit den Faeces wurden 4 % der verabreichten Dosis ausgeschieden, das entspricht 14,8 % der resorbierten Radioaktivität. Nach 168 Stunden verblieben 3 % der verabreichten Menge im Körper, wobei der größte Anteil in der Haut detektiert wurde. Die Resorptionskonstante (k [h^–1^]) für die Aufnahme ins Plasma betrug 0,14–0,21 pro Stunde. Die Halbwertszeiten der Elimination betrugen 3,39 Stunden und 4,42 Stunden in der schnellen Phase sowie 188 und 109 Stunden in der langsamen Phase. Daraus lässt sich schließen, dass eine Akkumulation von EDAO bei wiederholter Exposition nicht auftreten würde. Die höchste Konzentration im Plasma (C_max_) wurde 2–3 Stunden nach der Applikation erreicht (The Dow Chemical Company 2008 a).

### 3.2 Metabolismus

Nach oraler und dermaler Gabe wurde EDAO vollständig hydrolysiert. Neben Formaldehyd wurde 2-Amino-2-ethyl-1,3-propandiol als einziger Metabolit mit mehr als 5 % der verabreichten Dosis in allen analysierten Urin- und Stuhlproben identifiziert. Vier weitere Metaboliten wurden nicht identifiziert (Abschnitt 3.1; The Dow Chemical Company 2008 a).

Nach 15-tägiger oraler Gabe wurden im Vergleich zur einmaligen Gabe keine veränderten Verweildauern und kein verändertes Verhältnis von Radioaktivität in Urin und Faeces beobachtet. Dies zeigt, dass der eigene Metabolismus nicht induziert wurde (The Dow Chemical Company 2008 a).

## 4 Erfahrungen beim Menschen

Zur wiederholten Exposition, Wirkung auf Haut und Schleimhäute, Reproduktionstoxizität, Genotoxizität und Kanzerogenität liegen keine Daten vor.

### Einmalige Exposition

Der Geruch des EDAOs wird als beißend beschrieben (IFA 2023 b).

### Allergene Wirkung

#### Hautsensibilisierende Wirkung

EDAO wurde in den DKG-Standardreihen „Kühlschmierstoffe“ und „Industrielle Biozide“ als 1%ige Testzubereitung in Vaseline getestet. Vorwiegend handelt es sich bei den mit EDAO getesteten Personen um Metallarbeiter mit Verdacht auf eine beruflich bedingte Kontaktdermatitis durch Kühlschmierstoffe. Seit dem letzten Nachtrag (Greim 2004) liegen 14 neue Studien vor (Tabelle 3), unter denen teilweise Kollektivüberschneidungen möglich sind. Bei der Bewertung der Ergebnisse ist zu bedenken, dass Epikutantest-Ergebnisse mit Formaldehyd sowie Formaldehydabspaltern wie EDAO insgesamt oftmals schwache und schlecht reproduzierbare Ergebnisse liefern (Greim 2004, siehe auch Tabelle 3). Oftmals fehlen Angaben, die eine genaue Zuordnung zum verwendeten Kühlschmierstoff ermöglichen. Zahlreiche Studien mit unterschiedlich großen Kollektiven ergaben eine deutlich erhöhte Reaktionsquote bei Metallarbeitern (bis zu 3,3 %, siehe Tabelle 3). Die hohe Reaktionsquote auf EDAO kann teilweise auch durch freigesetztes Formaldehyd bedingt sein. Dennoch zeigt sich keine vollständige Übereinstimmung der Testreaktionen auf EDAO und Formaldehyd (beispielsweise Geier et al. 2013; Schubert und Geier 2021).

**Tab. 3:** Hautreaktionen auf EDAO in Epikutantests bei Personen mit Verdacht auf Kontaktallergie auf Kühlschmierstoffe seit 2002

**getestete Personen**	**Konzentration, Vehikel**	**Ergebnis:** **Reaktion bei**	**Bemerkungen**	**Literatur**
134	1 % in Vaseline	4 von 134 (3 %)	**Testzeitraum**: 1992–2003; **Kollektiv**: Von 5123 Personen, die im Zeitraum getestet wurden, waren 268 Metallarbeiter mit Verdacht auf beruflich bedingte Kontaktdermatitis auf Kühlschmierstoffe. 141 dieser Personen (7 ♀, 134 ♂, 18–60 Jahre (Median 39 Jahre)) wurden mit Proben der verwendeten Kühlschmierstoffe getestet, davon 28 Personen mit vergangener oder aktueller atopischer Dermatitis. 134 wurden mit EDAO getestet.	Geier et al. 2004 c
davon		2 von 27 (7,4 %)	Alle 27 Personen mit positiver Reaktion auf das verwendete wasserbasierte Kühlschmiermittel (k. w. A.) wurden auf EDAO getestet.	
		2 von 107 (1,9 %)	Von 114 Personen ohne positive Reaktion auf das verwendete wasserbasierte Kühlschmiermittel (k. w. A) wurden 107 mit EDAO getestet. **Ablesezeitpunkt und Ergebnis**: (D2) und D3, k. A. über Reaktionsstärke, OR: 4,2; 95-%-KI: 0,3–59,7	
106	1 % in Vaseline	1 von 106 (0,9 %)	**Testzeitraum**: 1999–2001; **Kollektiv**: IVDK-Studie, von insgesamt 20 695 Personen, die im Testzeitraum untersucht wurden, hatten 1842 Personen Berufsdermatitis, davon 160 Metallarbeiter (9 ♀, 151 ♂, Median 39 Jahre), die gegen wasserbasierte Kühlschmierstoffe exponiert waren. Davon wurden 106 mit EDAO getestet. **Ablesezeitpunkt und Ergebnis**: D3, k. A. über Reaktionsstärke, vermutlich Kollektivüberschneidung mit Geier et al. 2004 c	Geier et al. 2003, 2004 b
197	1 % in Vaseline	5 von 197 (2,5 %)	**Testzeitraum**: 2002–2003; **Kollektiv**: IVDK-Auswertung, von insgesamt 16 848 im Testzeitraum untersuchten Personen waren 251 Metallarbeiter (20 ♀, 231 ♂, Median: 38 Jahre) mit Verdacht auf Allergie gegen Kühlschmierstoffe. Davon wurden 197 mit EDAO getestet. **Ablesezeitpunkt und Ergebnis**: D3, 2 × ? (k. A. über Reaktion auf Formaldehyd), 3 × 1+ (davon 2 mit 1+ Reaktion auf Formaldehyd), 2 × 2+ (beide negativ auf Formaldehyd), % positiv (95-%-KI) 2,5 (2,1–8,5), vermutlich Kollektivüberschneidung mit Geier et al. 2004 c	Geier et al. 2004 a
139	1 % in Vaseline	3 von 139 (2,9 %)	**Testzeitraum**: 2002–2003; **Kollektiv**: IVDK-Auswertung von 144 Metallarbeitern (9 ♀, 135 ♂, durchschn. 40 Jahre) mit Exposition gegen Kühlschmierstoffe, wobei 10 frühere Metallarbeiter waren (keine aktuelle Exposition), bei 111 Personen wurde arbeitsplatzbezogene Dermatitis diagnostiziert. Es wurden 139 Personen mit EDAO getestet. **Ablesezeitpunkt und Ergebnis**: D3 (D4, falls D3 nicht möglich), 1 × ?, 2 × 1+, 1 × 2+, (zusätzlich positive Reaktion auf Methylenbismorpholin), vermutlich Kollektivüberschneidung mit Geier et al. 2004 c und Geier et al. 2004 a	Geier et al. 2006
739	1 % in Vaseline	21 von 739 (2,8 %)	**Testzeitraum**: 2005–2009; **Kollektiv**: IVDK-Auswertung von insgesamt 52 264 im Testzeitraum untersuchten Personen, davon waren 803 Metallarbeiter (59 ♀, 744 ♂, 56 % über 40 Jahre alt) mit vermutetem Kontaktekzem durch Kühlschmierstoffe, davon wurden 739 mit EDAO getestet. **Ablesezeitpunkt und Ergebnis**: D3 (D4), 13 × ?, 14 × 1+, 6 × 2+, 1 × 3+, 3 × irr., bei 678 dieser Personen parallele Testung mit EDAO und Formaldehyd: von 17 (2,5 %) mit positiver Reaktion auf EDAO reagierten 5 (30 %) auch positiv auf Formaldehyd	Geier et al. 2013
k. A.	1 % in Vaseline	2,5 %	**Testzeitraum**: 2014–2017; **Kollektiv**: IVDK-Auswertung der Epikutantestergebnisse von 861 Metallbearbeitern mit Verdacht auf Kühlschmierstoff-Allergie, k. A. über die Anzahl der mit EDAO getesteten Personen, **Ablesezeitpunkt und Ergebnis**: k. A. zu Ablesezeitpunkt, Reaktionsstärken	Geier 2019
2003	1 % in Vaseline	40 von 2003 (2,0 %)	**Testzeitraum**: 2010–2018; **Kollektiv**: IVDK-Auswertung von insgesamt 107 228 Personen. Von 17 952 Personen mit Berufsdermatose waren 3356 Metallarbeiter (349 ♀, 3007 ♂, überwiegend 20–60 Jahre), davon 804 mit täglichem Kontakt zu Kühlschmierstoffen (Zerspanungsmechaniker, d. h. Dreher, Bohrer und Werkzeugmacher), 2197 mit gelegentlichem Kontakt (Maschinisten, Mechaniker etc.) und 355 ohne Kontakt (z. B. Feinmechaniker, Schweißer etc.). Insgesamt wurden 2003 von ihnen im Rahmen der Kühlschmierstoff-Reihe mit EDAO getestet. **Ablesezeitpunkt und Ergebnis**: D3 (D4, falls D3 nicht möglich), Ergebnisse in Abhängigkeit von Häufigkeit des Kontakts mit Kühlschmierstoffen	Schubert et al. 2020; Schubert und Geier 2021
davon		20 von 681 (2,9 %)	mit häufigem Kontakt (95-%-KI 1,7–4,4),	
		19 von 1181 (1,6 %)	mit gelegentlichem Kontakt (95-%-KI 0,9–2,4),	
		1 von 141 (0,7 %)	ohne Kontakt (95-%-KI 0,0–3,9),	
			Von 674 Personen, die mit EDAO getestet wurden, reagierten 31 (4,6 %) positiv auf Formaldehyd (1 % in Wasser), 20 (3,0 %) positiv auf EDAO (1 % in Vaseline) und 10 sowohl positiv auf Formaldehyd als auch auf EDAO. Vermutlich Kollektivüberschneidung mit Geier 2019	
210	1 % in Vaseline	13 von 210 (6,2 %)	**Testzeitraum**: 2004–2005; **Kollektiv**: Retrospektive Auswertung der Daten von 210 Personen mit positiver Reaktion auf Formaldehyd oder einen Formaldehydabspalter. Es reagierten 24 (11 %) positiv auf Formaldehyd, EDAO (Bioban CS-1246), 4,4-Dimethyloxazolidin (Bioban CS-1135) sowie ein Gemisch aus 4-(2-Nitrobutyl)morpholin und 4,4′-(2-Ethyl-2-nitrotrimethylen)dimorpholin (Bioban CX-1487) oder andere Formaldehydabspalter. **Ablesezeitpunkt und Ergebnis**: D3 und D4/5, Reaktionsstärken: 1 × ± (Masseur), 11 × 1+ (2 Maschinenführer, 1 LKW-Mechaniker, 2 Krankenpflegerinnen, 4 nicht berufstätig, 2 Rentner), 2 × 2+ (Schifffahrtskaufmann/-frau, Industriemaschinenreparateur/in), Von insgesamt 15 Personen, die positiv auf Biobane (darunter EDAO) reagierten, reagierte eine Person ausschließlich auf 4,4-Dimethyloxazolidin, 11 Personen auch auf Formaldehyd und 14 Personen auch auf einen anderen Formaldehydabspalter. Von 17 Personen, die insgesamt positiv auf Formaldehyd reagierten, reagierten 11 auch auf eines der getesteten Biobane.	Anderson et al. 2007
k. A.	k. A., vermutlich 1 % in Vaseline	2 von k. A.	**Testzeitraum**: 2010–2011; **Kollektiv**: Aus einem Gesamtkollektiv von 316 Personen mit Verdacht auf beruflich bedingte Kontaktallergie wurden 228 (145 ♀, durchschn. 36 Jahre; 83 ♂, durchschn. 41 Jahre) Personen untersucht, 110 davon mit diagnostizierter beruflich bedingter allergischer Kontaktdermatitis (ACD) (andere: keine berufliche ACD oder kein Epikutantest durchgeführt). 19 Personen wurden mit verschiedenen Konservierungsstoffen, darunter EDAO getestet. Es wurde über 2 positive Reaktionen auf EDAO berichtet, jedoch bleibt unklar, wieviel Personen mit EDAO getestet worden sind (zwischen 2 und 19). **Ablesezeitpunkt und Ergebnis**: D2, D3-D4, D7, k. A. zur Reaktionsstärke, k. A. zu weiteren pos. Reaktionen	Friis et al. 2013
24	1 % in Vaseline	5 von 24	**Testzeitraum**: 2001–2007; **Kollektiv**: Von 1166 Personen, die mit Standardreihen getestet wurden, wurde bei 81 (6,9 %) eine Formaldehyd-Allergie identifiziert (es reagierten 71 (6,1 %) auf 1 % Formaldehyd, bei weiteren 9 war bereits eine Formaldehyd-Allergie bekannt, eine Person reagierte auf 2 % Formaldehyd). Von diesen positiv auf Formaldehyd reagierenden Personen wurden 24 Personen im Rahmen der Öl- und Schmierstoffreihe mit EDAO getestet. **Ablesezeitpunkt und Ergebnis**: D2, (D3) und D4/5/6, Von 11 Personen mit neuer beruflich bedingter allergischer Kontaktdermatitis gegen Formaldehyd reagierten 4 positiv, davon: 2 × 2+ (Mechaniker, Auszubildender zum Mechaniker), 1 × 1+ (Bohrarbeiter), 1 × ?+ (Monteur), Die Angabe der Reaktionsstärke einer weiteren positiv getesteten Person, bei der die Formaldehyd-Allergie bereits zu einem früheren Zeitpunkt diagnostiziert wurde, fehlt.	Aalto-Korte et al. 2008
367	1 % in Vaseline	12 von 367 (3,3 %)	**Testzeitraum**: 2007–2020; **Kollektiv**: Die Daten von 1497 Personen, die mit Standardreihen getestet wurden, wurden retrospektiv auf positive Reaktionen auf Formaldehyd und Formaldehydabspalter ausgewertet. 93 Personen reagierten positiv auf Formaldehyd (Testzubereitung: 2 %; 1 %; 0,32 %; 0,1 % wässrig), davon 74 gleichzeitig positiv auf mindestens einen Formaldehydabspalter. Es gab 9 Formaldehyd-unabhängige Reaktionen auf einen Formaldehydabspalter. Insbesondere Personen mit 3+-Reaktion auf Formaldehyd reagierten häufiger auf einen Formaldehydabspalter als Personen mit schwächerer Reaktion auf Formaldehyd. 367 Personen wurden in der Öl- und Schmierstoff-Reihe mit EDAO getestet. Davon reagierten 104 positiv auf Formaldehyd oder einen Formaldehydabspalter. **Ablesezeitpunkt und Ergebnis**: Es wurde 2–3 × abgelesen: D2-D3-D4, D2-D3-D6, oder D2-D5, 12 Personen reagierten positiv, davon wurden 11 (92 %) auch positiv auf Formaldehyd getestet. Angaben zur Reaktionsstärke auf EDAO fehlen.	Aalto-Korte und Pesonen 2021
175	1 % in Vaseline	2 von 175 (1,1 %)	**Testzeitraum**: 1999–2003; **Kollektiv**: 175 Personen (2 ♀, 173 ♂, 19–63 Jahre) mit Verdacht auf beruflich bedingte Kontaktdermatitis im Zusammenhang mit Ölen und Kühlschmierstoffen (Israel); **Ablesezeitpunkt und Ergebnis**: D2 und D3, k. A. zur Reaktionsstärke	Trattner et al. 2009
k. A.	k. A.	6,2 %	**Testzeitraum**: k. A.; **Kollektiv**: unselektiertes Kollektiv, USA; **Ablesezeitpunkt und Ergebnis**: k. w. A.	zitiert in de Groot und Flyvholm 2020

1+, 2+, 3+: Stärke der Hautreaktion im Epikutantest; ?: unklar, ob allergisch oder irritativ; D: Tag nach Applikation; durchschn.: durchschnittlich; irr.: irritativ; k. A.: keine Angabe; KI: Konfidenzintervall; OR: Odds Ratio; pos.: positiv

Weiterhin wird über den Fall eines Metallarbeiters (39 Jahre) mit über einem Jahr bestehender arbeitsplatzbezogener Dermatitis an den Handrücken und Unterarmen und Kontakt zu Kühlschmierstoffen berichtet. Im Epikutantest reagierte er mit einer fraglichen Reaktion am 3. und 4. Tag nach der Applikation auf EDAO (1 % in Vaseline) und auf die verwendeten Kühlschmiermittel ebenfalls mit fraglichen Reaktionen. Auf Formaldehyd reagierte die Person erst bei Nachtestung mit einer 2+-Reaktion am 3. Tag. Die Autoren folgern, dass eine Allergie gegen Formaldehyd verantwortlich für die fraglich oder schwach positiven Reaktionen auf Formaldehydabspalter war (Geier et al. 2008).

## 5 Tierexperimentelle Befunde und In-vitro-Untersuchungen

### 5.1 Akute Toxizität

#### 5.1.1 Inhalative Aufnahme

Hierzu liegen keine neuen Daten vor.

#### 5.1.2 Orale Aufnahme

Hierzu liegen keine neuen Daten vor.

#### 5.1.3 Dermale Aufnahme

Nach OECD-Prüfrichtlinie 402 wurden bei je fünf männlichen und weiblichen Wistar-Ratten 2000 mg unverdünntes EDAO/kg KG (1,85 ml/kg KG, Reinheit 98,7 %) okklusiv auf die rasierte Haut aufgetragen. Nach 24 Stunden wurde die Substanz abgewaschen und die Haut 14 Tage beobachtet. Es traten keine lokalen Hautreaktionen auf. Jedoch wurde eine Überempfindlichkeit der männlichen und weiblichen Ratten bei der Handhabung beobachtet, die zwei bis drei Stunden andauerte. Die LD_50_ ist damit bei Ratten > 2000 mg/kg KG (Advinus Therapeutics Pvt Ltd 2009).

Nach 24-stündiger okklusiver Auftragung von 0, 500, 1000, 1500 oder 2000 mg unverdünntes EDAO/kg KG auf die intakte oder skarifizierte Haut von je zwei bis vier Kaninchen war eine starke Reizwirkung bei allen behandelten Tieren zu verzeichnen. Erytheme und Ödeme blieben während einer 14-tägigen Nachbeobachtung bestehen. Es wurde eine LD_50_ von 1948 mg/kg KG berechnet. Außer niedrigeren Körpergewichten bei den überlebenden Tieren der höchsten Dosis wurden keine weiteren Effekte beobachtet (Greim 1993).

### 5.2 Subakute, subchronische und chronische Toxizität

#### 5.2.1 Inhalative Aufnahme

Hierzu liegen keine Daten vor.

#### 5.2.2 Orale Aufnahme

Die Daten der Studien mit EDAO sind in Tabelle 4 dargestellt.

##### 5.2.2.1 Ratte

Nach siebentägiger Gabe von 0, 10, 100, 500 oder 1000 mg EDAO/kg KG und Tag in Kapseln an männliche Sprague-Dawley-Ratten traten keine systemischen Effekte auf. Die Dosen von 500 und 1000 mg/kg KG führten zu einer geringeren Nahrungsaufnahme (k. w. A.; ECHA 2013).

In einer Schlundsondenstudie nach OECD-Prüfrichtlinie 407 erhielten je fünf weibliche und männliche SD-Ratten pro Dosisgruppe an 28 Tagen täglich 0, 100, 300 oder 1000 mg EDAO/kg KG und Tag in Wasser. Ab 300 mg/kg KG wurden Reizungen und Entzündungen der Magenschleimhaut beobachtet (ECHA 2013; Greim 1993). Es ist wahrscheinlich, dass die Reizungen auch auf die Zunahme des pH-Wertes durch das Hydrolyseprodukt 2-Amino-2-ethyl-1,3-propandiol zurückzuführen sind.

In einer weiteren 28-Tage-Schlundsondenstudie nach OECD-Prüfrichtlinie 407 mit täglicher Gabe an je fünf weibliche und männliche Crl:CD(SD)-Ratten pro Dosisgruppe von 0, 50, 100, 300 oder 1000 mg EDAO/kg KG und Tag (Reinheit 98,55 %) in Maiskeimöl traten bereits ab der niedrigsten eingesetzten Dosis Hyperplasien an der Magenschleimhaut auf. Dies verstärkte sich mit zunehmender Dosis zu Entzündungen sowie erosiven und ulzerativen Effekten. Das relative Lebergewicht der Tiere der höchsten Dosisgruppe war signifikant erhöht. Alle weiteren adversen Effekte waren Folgen der Reizungen oder Entzündungen der Magenschleimhaut (The Dow Chemical Company 2006).

Nach täglicher Schlundsondengabe von 0, 10, 50 oder 250 mg EDAO/kg KG und Tag (Reinheit 98,55 %) in Maiskeimöl nach OECD-Prüfrichtlinie 408 an je zehn weibliche und männliche Crl:CD(SD)-Ratten pro Dosisgruppe über einen Zeitraum von 90 Tagen wurden ab 50 mg/kg KG Entzündungen der Magenschleimhaut und weitere Effekte am Magen beobachtet. Auch nach einer 28-tägigen Erholungszeit zeigten sich noch Entzündungen an der Magenschleimhaut. Bei den männlichen Tieren der höchsten Dosis ergab die mikroskopische Untersuchung Eosinophilie in den zentrilobulären Hepatozyten. Die Autoren leiten für die lokale Reizwirkung im Gastrointestinaltrakt einen NOAEL von 10 mg/kg KG und Tag ab (The Dow Chemical Company 2007 d).

In einer nach OECD-Prüfrichtlinie 416 durchgeführten 2-Generationenstudie mit zwölfwöchiger Schlundsondengabe lag der NOAEL für Parentaltoxizität bei 5 mg EDAO/kg KG und Tag. Die genauen Daten sind in Abschnitt 5.5.1 beschrieben (The Dow Chemical Company 2008 b).

###### 5.2.2.1.1 Hydrolyseprodukt 2-Amino-2-ethyl-1,3-propandiol

In einer 90-tägigen Studie nach OECD-Prüfrichtlinie 408 mit Schlundsondengabe erhielten je zehn weibliche und männliche Wistar-Han-Ratten pro Dosisgruppe 0, 100, 300 oder 1000 mg 2-Amino-2-ethyl-1,3-propandiol/kg KG und Tag (Reinheit 92,80 %) in ultrareinem Wasser. Ab 300 mg 2-Amino-2-ethyl-1,3-propandiol/kg KG und Tag kam es im Magen vereinzelt zu gemischten Zellinfiltrationen und Ödemen der Submukosa des Drüsenmagens sowie zu Effekten im mesenterialen Lymphknoten (Erythrophagozytose, dunkelrote Verfärbung), was auf eine lokale Entzündung im Magen hindeutet. Bis zur höchsten Dosis von 1000 mg/kg KG und Tag traten keine systemischen Effekte (einschließlich ophthalmologischer Untersuchung und „functional observational battery“) auf (Charles River Laboratories 2024 a).

In einer kombinierten Studie zur Untersuchung der Toxizität und Reproduktionstoxizität mit wiederholter oraler Verabreichung nach der OECD-Prüfrichtlinie 422 (siehe Abschnitt 5.5) wurde je 70 männlichen und weiblichen Sprague-Dawley-Ratten (Crj:CD(SD)IGS) 0, 250, 500 oder 1000 mg 2-Amino-2-ethyl-1,3-propandiol/kg KG und Tag (Reinheit 99,4 %) in ultrareinem Wasser per Schlundsonde verabreicht. Die Tiere wurden 14 Tage vor und während der Verpaarung behandelt. Für die männlichen Tiere betrug die Expositionszeit bis zum Untersuchungszeitpunkt 42 Tage und für die weiblichen Tiere, die während der Gestation sowie bis zum vierten Postnataltag behandelt wurden, 42 bis 48 Tage. Bei der höchsten Dosis von 1000 mg 2-Amino-2-ethyl-1,3-propandiol/kg KG und Tag kam es bei den männlichen Ratten zu entzündlichen Veränderungen an Vor- und Drüsenmagen sowie weiteren Effekten am Magen, die für eine lokale Reizwirkung sprechen. Bei dieser Dosis trat bei den männlichen Tieren eine erniedrigte Anzahl an Aufstehversuchen in der 3., 4., 5. und 6. Behandlungswoche auf. Am Ende der Erholungszeit war der Effekt nicht mehr nachweisbar (Bozo Research Center Co 2004).

##### 5.2.2.2 Hund

Eine 28-Tage-Schlundsondenstudie wurde mit je zwei männlichen und weiblichen Beagle-Hunden pro Dosisgruppe durchgeführt. Nach Gabe von 0; 1,5; 5 oder 15 mg EDAO/kg KG und Tag in Maiskeimöl trat in der höchsten Dosisgruppe Erbrechen bei drei Tieren auf. Die Abnahme des absoluten und relativen Thymusgewichtes der Tiere der höchsten Dosisgruppe korrespondierte nicht mit histopathologischen Veränderungen. Die Augeneffekte bei den Tieren der 5-mg/kg-Gruppe wurden von den Autoren als spontan auftretend bewertet. Die mikroskopische Untersuchung zeigte keine Reizwirkung an der Magenschleimhaut. Körpergewichte, Nahrungsaufnahme und Gewichte ausgewählter Organe waren unverändert. Die Autoren leiten aus den Ergebnissen einen NOAEL von 15 mg/kg KG ab (The Dow Chemical Company 2007 b).

Je vier männlichen und vier weiblichen Beagle-Hunden pro Gruppe wurden täglich 0; 1,5; 5 oder 15 mg EDAO/kg KG und Tag in Maiskeimöl per Schlundsonde 90 Tage lang verabreicht (OECD-Prüfrichtlinie 409). Es trat in allen Dosisgruppen Erbrechen (einmal während der 90 Tage, nach 15 mg/kg KG zwei- bis dreimal) auf, das von den Autoren auf Übelkeit durch die EDAO-Gabe und nicht auf Magenreizungen zurückgeführt wurde. Die mikroskopische Untersuchung zeigte keine Reizwirkung an der Magenschleimhaut. Weibliche Tiere der Hochdosisgruppe hatten einen leicht erhöhten Urin-pH-Wert, begleitet von Phosphatkristallen. Da keine dazugehörigen histopathologischen Effekte beobachtet wurden, sehen die Autoren dies zwar als substanzbedingt, aber nicht als advers an. Die Autoren leiten aus den Ergebnissen einen NOAEL von 15 mg/kg KG und Tag ab (The Dow Chemical Company 2007 c).

**Tab. 4:** Toxizität von EDAO nach wiederholter oraler Verabreichung

**Spezies, ** **Stamm, ** **Anzahl pro Gruppe**	**Exposition**	**Befunde^a)^**	**Literatur**
Ratte, Sprague Dawley, ♂, k. w. A.	7 d, 0, 10, 100, 500, 1000 mg EDAO/kg KG und Tag, Kapselgabe	**100 mg/kg KG**: **NOAEL**; **ab 500 mg/kg KG**: ♂: Nahrungsaufnahme ↓	ECHA 2013
Ratte, Sprague Dawley, 5 ♂, 5 ♀	28 d, 0, 100, 300, 1000 mg/kg KG und Tag, Schlundsonde, in Wasser, Reinheit k. A., OECD TG 407	**100 mg/kg KG**: **NOAEL**; **ab 300 mg/kg KG**: ♂/♀: Magenschleimhaut: Reizungen, Entzündungen; **1000 mg/kg KG**: ♂/♀: KG-Zunahme ↓, Nahrungsaufnahme ↓, ♂: Salivation, Piloarrektion, Nierengewicht ↑	ECHA 2013, Greim 1993
Ratte, Sprague Dawley, 5 ♂, 5 ♀	28 d, 0, 50, 100, 300, 1000 mg/kg KG und Tag, Schlundsonde, in Maiskeimöl, 7 d/Wo, Reinheit 98,55 %, OECD TG 407	**50 mg/kg KG**: **LOAEL**; **ab 50 mg/kg KG**: Größe der Grenzfalte des Magens ↑ (♂: 2/5, ♀: 3/5), Magen: Hyperplasien der Grenzfalte sehr gering bis gering (♂: 4/5, ♀: 3/5), Magenschleimhaut: multifokale oder diffuse Hyperplasien u. Hypertrophien sehr gering (♂: 1/5, ♀: 3/5), ♂: Leber: Makrophagenaggregation u. Vakuolisierung sehr gering; **ab 100 mg/kg KG**: ♂/♀: Salivation, Größe der Grenzfalte des Magens ↑ (♂: 5/5, ♀: 4/5), Magen: Hyperplasien der Grenzfalte gering–mäßig (♂: 5/5, ♀: 4/5); **ab 300 mg/kg KG**: Größe der Grenzfalte des Magens ↑ (♂: 5/5, ♀: 5/5), Magen: Hyperplasien der Grenzfalte sehr gering–gering (♂: 4/5, ♀: 3/5), Entzündungen der Submucosa gering (♀: 4/5), glanduläre Magenschleimhaut: Entzündungen mäßig (♂: 1/5), Erosion gering (♂: 1/5, ♀: 2/5), Pylorus: Erosion (♂: 3/5, ♀: 2/5); ♂/♀: Serum: AST ↑, Blutplättchen ↑, ♀: Retikulozyten ↑, ♂: Leukozyten ↑, Prothrombinzeit ↓, **1000 mg/kg KG**: ♂/♀: KG ↓, KG-Zunahme ↓, Geschwüre der glandulären u. nicht-glandulären Magenschleimhaut (♂: 4/5, ♀: 5/5), Größe der Grenzfalte des Magens ↑ (♂: 2/5, ♀: 1/5), raue Oberfläche der nicht-glandulären Magenschleimhaut (♂: 4/5, ♀: 4/5), Blut: Retikulozyten ↑, Leukozyten ↑, Serum: ALT ↑, Gesamtprotein ↓, Albumin ↑, Glucose ↓, Triglyceride ↑, Magen u. Magenschleimhaut: Hyperkeratose (♂: 5/5, ♀: 4/5), Hyperplasie (♂: 4/5, ♀: 2/5), Entzündungen gering–mäßig (♂: 5/5, ♀: 4/5), Pylorus: Erosionen sehr gering bis gering (♂: 3/5, ♀: 3/5), Entzündungen gering (♀: 1/5), Leber: rel. Gewicht ↑ (♂: 11 %, ♀: 8 %), ♂: Nahrungsaufnahme ↓, Gehirn rel. Gew. ↓, Testes rel. Gew. ↓, Epididymides rel. Gew. ↓, Blut: hypochrome mikrozytäre Anämie, Erythrozyten: Polychromasie, Hypochromie u. verringerte Größe, Neutrophile ↑, Lymphozyten ↓, Monozyten ↑, zentrilobuläre Hypertrophie, Duodenum: Hyperplasie u. Hypertrophie der Mucosa	The Dow Chemical Company 2006
Ratte, Sprague Dawley, 10 ♂, 10 ♀	90 d, 0, 10, 50, 250 mg/kg KG und Tag, Schlundsonde, in Maiskeimöl, 7 d/Wo, 28 d Nachbeobachtung, Reinheit 98,55 %, OECD TG 408	**10 mg/kg KG**: **NOAEL**; **ab 50 mg/kg KG**: Verdickungen der Grenzfalte des Magens (♂: 10/10, ♀: 8/10), Magen: Hyperplasien Grenzfalten-Plattenepithel (♂: gering 10/10, ♀: sehr gering bis gering 8/10), Entzündungen der Submucosa: fokal sehr gering (♂: 2/10, ♀: 1/10), multifokal sehr gering (♀: 2/10), glanduläre Magenschleimhaut: Entzündungen: sehr gering (♀: 1/10), Hypertrophie sehr gering (♂: 2/10); **250 mg/kg KG**: Magen: ♀: Hyperplasien Grenzfalte-Plattenepithel gering (10/10), Entzündungen der Submucosa multifokal sehr gering bis mäßig (♂: 10/10, ♀: 10/10), glanduläre Magenschleimhaut: Hypertrophie gering (♂: 10/10, ♀: 10/10), Entzündungen multifokal sehr gering bis gering (♂: 9/10, ♀: 10/10), Mitosezahl ↑ (♂: 5/10), Erosionen multifokal (♂: 1/10), fokale Nekrose (♀: 1/10), ♂/♀: Salivation, Retikulozyten ↑, ♂: Cholesterin ↓, Geschwür an der glandulären Magenschleimhaut (1/10), Leber: Eosinophilie sehr gering (♂: 8/10); ♀: Urin: Blut (9/10), Verdickungen der Grenzfalte des Magens (10/10), **28 d Nachbeobachtung**: **ab 50 mg/kg KG**: Magen: Entzündungen der Submucosa fokal sehr gering (♂: 3/10, ♀: 2/10), multifokal sehr gering (♂: 1/10); **250 mg/kg KG**: Magen: Entzündungen der Submucosa multifokal sehr gering (♂: 10/10, ♀: 9/10)	The Dow Chemical Company 2007 d
Hund, Beagle, 2 ♂, 2 ♀	28 d, 0; 1,5; 5; 15 mg/kg KG und Tag, Schlundsonde, in Maiskeimöl, 7 d/Wo, Reinheit 98,55 %	**5 mg/kg KG**: **NOAEL**; **15 mg/kg KG**: Erbrechen (♂: 1/2, ♀: 2/2), ♂/♀: Thymus: abs. u. rel. Gew. ↓, ♀: Urin: Blut (stark, 2/2)	The Dow Chemical Company 2007 b
Hund, Beagle, 4 ♂, 4 ♀	90 d, 0; 1,5; 5; 15 mg/kg KG und Tag, Schlundsonde, in Maiskeimöl, 7 d/Wo, Reinheit 98,55 %, OECD TG 409	**1,5 mg/kg KG**: Erbrechen (♂: 1/4, ♀: 1/4); **5 mg/kg KG**: ♀: Erbrechen (1/4); **15 mg/kg KG**: Erbrechen (♂: 3/4, ♀:2/4), ♂: Urin: Blut (gering, 3/4)	The Dow Chemical Company 2007 c

^a)^ wenn nicht anders angegeben, sind die aufgeführten Veränderungen statistisch signifikant

a: Jahr; abs: absolut; ALT: Alaninaminotransferase; AST: Aspartataminotransferase; d: Tag; Gew.: Gewicht; h: Stunde; k. A.: keine Angabe; Mo: Monat; rel.: relativ; TG: Test Guideline (Prüfrichtlinie); u.: und; Wo: Woche

#### 5.2.3 Dermale Aufnahme

Je sechs weibliche und männliche Wistar-Ratten wurden sechs Stunden am Tag, fünf Tage pro Woche, drei Wochen lang dermal gegen 0, 30, 100 oder 300 mg EDAO/kg KG (Reinheit 96 %, 0; 0,75; 1,25 oder 3,75 % (G/V) in Wasser) exponiert. Es traten lokale Hautreizungen auf, die sich dosisabhängig steigerten. Nekrotische Erytheme, Schorf- und Narbenbildung traten in den beiden hohen Dosisgruppen auf. Bei der niedrigsten Dosis konnten Erytheme und bei zwei Tieren Schorfbildung beobachtet werden. Weibliche Tiere der hohen Dosisgruppe hatten einen statistisch signifikanten Anstieg des Nieren- und Körpergewichts. Die Untersuchung der hämatologischen und klinischen Parameter ergaben keinen positiven Befund (Greim 1993; The Dow Chemical Company 2007 a).

### 5.3 Wirkung auf Haut und Schleimhäute

Hier werden die Daten des EDAOs und des Hydrolyseprodukts 2-Amino-2-ethyl-1,3-propandiol dargestellt.

2-Amino-2-ethyl-1,3-propandiol hat einen pH-Wert von 10,8 (bei 0,1 M) (IFA 2023 a) und einen pKs-Wert von 9,03 (ECHA 2018).

#### 5.3.1 Haut

Sofort nach dem Ende einer vierstündigen okklusiven Applikation von 0,5 ml EDAO kam es nicht zu Hauteffekten, während nach einer Nachbeobachtungszeit von 24 Stunden Erytheme und leichte Ödeme bei fünf von sechs Kaninchen auftraten. Nach 48 Stunden bestanden die Effekte noch bei zwei Tieren. Die Autoren schließen daraus, dass EDAO keine ätzende Wirkung an der Haut zeigt (Greim 1993).

Nach 24-stündiger okklusiver Applikation von 0, 500, 1000, 1500 oder 2000 mg EDAO/kg KG auf die intakte oder skarifizierte Haut von je zwei bis vier Kaninchen war eine schwere Reizwirkung bei allen behandelten Tieren zu verzeichnen. Erytheme und Ödeme waren bis zum Ende der 14-tägigen Nachbeobachtung vorhanden und bei den höheren Dosen auch Nekrosen (Greim 1993).

Nach 24-stündiger okklusiver dermaler Behandlung der intakten oder skarifizierten Kaninchenhaut mit 0,5 ml EDAO (unverdünnt) konnte ein Irritationsindex der intakten und skarifizierten Haut von 5,7 (Ablesezeitpunkte 24 und 72 Stunden nach Applikation, ausgewertet nach Draize) und damit eine starke Reizwirkung (Erytheme und Ödeme) festgestellt werden (Greim 1993; IMC 1980). Die Berechnung des Reizindexes nur für die intakte Haut nach 24 und 72 Stunden ergibt für alle Kaninchen einen gemittelten Reizwert von 5 (von 8).

Der Stoff ist nach dem global harmonisierten System von den REACH-Registranten in die Kategorie 2 für hautreizende Stoffe eingestuft (ECHA 2013).

**Fazit**: EDAO wirkt nach vierstündiger okklusiver Behandlung reizend, nach 24-stündiger okklusiver Applikation stark reizend an der Haut von Kaninchen.

##### Hydrolyseprodukt 2-Amino-2-ethyl-1,3-propandiol

Sechs Kaninchen erhielten 0,5 ml 2-Amino-2-ethyl-1,3-propandiol (unverdünnt, Reinheit 85,38 %) 24 Stunden lang okklusiv auf die intakte oder skarifizierte Haut. Die Befunderhebung erfolgte 24 und 72 Stunden nach Versuchsbeginn. 2-Amino-2-ethyl-1,3-propandiol bewirkte auf der intakten Haut leichte Erytheme sowie mäßige bis starke Ödeme. Diese Hautveränderungen waren innerhalb einer Woche reversibel. Die skarifizierte Haut zeigte bei allen Tieren starke Ödeme und Nekrosen, die noch nach 21 Tagen nachweisbar waren. Der primäre Reizwert für intakte und skarifizierte Haut betrug 4,6 (von maximal 8). 2-Amino-2-ethyl-1,3-propandiol wurde daher als stark reizend für die Kaninchenhaut bewertet (ECHA 2018). Nach dem gleichen Schema wie bei EDAO wurde der Reizindex nur für die intakte Haut nach 24 und 72 Stunden berechnet. Für alle Kaninchen ergibt sich ein mittlerer Reizwert von 2,3 (von 8).

Eine vierstündige okklusive Applikation von 0,5 ml 2-Amino-2-ethyl-1,3-propandiol (unverdünnt, Reinheit 85,34 %) auf die geschorene, intakte Haut führte nach 4, 24 und 48 Stunden bei keinem der sechs behandelten Kaninchen zu Erythemen oder Ödemen (ECHA 2018).

Da nach vierstündiger okklusiver Exposition keine Hautreizung auftrat, erfolgte von den REACH-Registranten keine Einstufung nach dem global harmonisierten System (ECHA 2018).

**Fazit**: 2-Amino-2-ethyl-1,3-propandiol wirkt nach vierstündiger okklusiver Applikation nicht reizend, nach 24-stündiger okklusiver Exposition reizend an der Haut von Kaninchen.

#### 5.3.2 Auge

EDAO verursachte starke Augenreizungen, die mindestens sieben Tage fortbestanden (Greim 1993). Der Stoff ist nach dem global harmonisierten System aufgrund schwerer Augenschäden von den REACH-Registranten in die Kategorie 1 eingestuft (ECHA 2013).

##### Hydrolyseprodukt 2-Amino-2-ethyl-1,3-propandiol

Neun Kaninchen wurde einmalig 0,1 ml 2-Amino-2-ethyl-1,3-propandiol (Reinheit 85 %) unverdünnt in jeweils einen Bindehautsack instilliert. Die Augen wurden 24, 48 und 72 Stunden nach der Behandlung untersucht. Bei allen Tieren zeigten sich nach 24 Stunden Hornhautläsionen und schwere Augenreizungen. Die Augen waren innerhalb von 14 Tagen keratinisiert. 2-Amino-2-ethyl-1,3-propandiol wirkte am Kaninchenauge ätzend (Greim 1995).

**Fazit**: 2-Amino-2-ethyl-1,3-propandiol wirkt ätzend am Auge, da es irreversible Schäden verursacht, und ist nach dem global harmonisierten System von den REACH-Registranten in die Kategorie 1 eingestuft (ECHA 2018).

### 5.4 Allergene Wirkung

#### 5.4.1 Hautsensibilisierende Wirkung

In den Nachträgen von 1996 und 2004 (Greim 1996, 2004) wurden bereits drei tierexperimentelle Untersuchungen am Meerschweinchen beschrieben, von denen eine ein positives Ergebnis lieferte. Es liegen drei neue Studien vor:

Ein unvollständig dokumentierter Bühler-Test an 20 Dunkin-Hartley-Meerschweinchen (k. w. A.) lieferte ein positives Ergebnis. Es erfolgten im Abstand von einer Woche zwei epikutane Applikationen einer 5%igen Testzubereitung (k. A. zur Reinheit) in Wasser, was die Hydrolyse von EDAO begünstigt. Die Provokationen mit 5- und 0,5%igen Testzubereitungen führten zu positiven Reaktionen, während eine Provokation mit einer 0,1%igen Testzubereitung ein negatives Ergebnis lieferte (ECHA 2013).

In einem Maximierungstest nach OECD-Prüfrichtlinie 406 an 20 Dunkin-Hartley-Meerschweinchen erfolgte die intradermale Injektion mit einer 5%igen und die topische Applikation mit einer 25%igen wässrigen Testzubereitung (k. A. zur Reinheit). Die erste Provokation fand mit einer 1%igen Testzubereitung statt, weitere am 6. Tag nach der ersten Provokation mit 0,2; 0,01 und 0,001%igen Testzubereitungen. Laut Aussage der Autoren bewirkten die Konzentrationen 0,001 % und 0,01 % eine schwache bzw. mäßige Reaktion (k. w. A.) und die 0,2%ige und 1%ige Testzubereitung eine starke bzw. extreme Reaktion (k. w. A.). Das Ergebnis wurde als positiv bewertet (ECHA 2013).

Bei einem zweiten Maximierungstest an zehn Dunkin-Hartley-Meerschweinchen erfolgte die intradermale Applikation mit einer 5%igen wässrigen Testzubereitung (Reinheit 98,8 %) und die topische Applikation mit einer 25%igen wässrigen Testzubereitung. Die Provokation bei fünf Tieren mit einer 1%igen Testzubereitung und bei fünf Tieren mit einer 0,5%igen Testzubereitung führte nicht zu einer positiven Reaktion, das Testergebnis ist damit als negativ zu bewerten (ECHA 2013). Die Bewertung dieses Ergebnisses gestaltet sich schwierig, da nur zehn Tiere getestet wurden. Negative Testergebnisse sollten jedoch laut OECD-Prüfrichtlinie 406 mit 20 Tieren pro Substanz untermauert werden.

**Fazit**: Trotz gleicher Bedingungen für die Induktion und Provokation wurden widersprüchliche Ergebnisse in diesen beiden Maximierungstests erhalten.

#### 5.4.2 Atemwegssensibilisierende Wirkung

Es liegen keine Untersuchungen vor.

### 5.5 Reproduktionstoxizität

#### 5.5.1 Fertilität

In einer 2-Generationenstudie nach OECD-Prüfrichtlinie 416 erhielten 27 Crl:CD(SD)-Ratten pro Geschlecht und Dosis 0, 5, 25 oder 150 mg EDAO/kg KG und Tag (Reinheit: 98,7 ± 0,01 %) per Schlundsonde (Vehikel: Maiskeimöl, 4 ml/kg KG, 7 Tage/Woche) zehn Wochen vor und während der Verpaarung sowie während Gestation und Laktation. Die Wahl der Dosierungen beruhte auf vorangegangenen Toxizitätsstudien. Bei 150 mg EDAO/kg KG und Tag wurden bei der Mehrheit der Tiere Anzeichen für eine Reizung des Magens festgestellt. Diese war makroskopisch gekennzeichnet durch eine Verdickung der nicht-glandulären Mucosa sowie histopathologisch durch eine Hyperplasie der Grenzfalte, einer subakut bis chronisch verlaufenden Entzündung der glandulären Mucosa und der Submucosa sowie durch Hyperplasie, Hypertrophie und erhöhte Mitosezahlen in der glandulären Mucosa. Zudem wurden bei dieser Dosis bei einigen weiblichen Tieren der F0-Generation und einigen adulten männlichen Tieren der F1-Generation Erosionen der glandulären Mucosa festgestellt. Auch bei 25 mg EDAO/kg KG und Tag kam es zu derartigen Effekten, jedoch in geringerer Häufigkeit und mit geringerem Schweregrad. Bei 150 mg EDAO/kg KG und Tag traten bei den männlichen Tieren der F0-Generation erhöhte absolute und relative Lebergewichte auf, die histopathologisch begleitet waren von geringer Eosinophilie der zentrilobulären Hepatozyten. Bei dieser Dosis kam es bei den männlichen Tieren der F0- und F1-Generation auch zu einer leichten behandlungsbedingten Zunahme der absoluten und relativen Schilddrüsengewichte ohne histopathologisches Korrelat. Während der Laktation wurde bei 150 mg EDAO/kg KG und Tag bei den weiblichen adulten Tieren der F0- und F1-Generation eine geringere Körpergewichtszunahme beobachtet. Bis zur höchsten Dosis war keine Beeinträchtigung der Fertilität und des Reproduktionsvermögens festzustellen. Der NOAEL für Parentaltoxizität liegt bei 5 mg EDAO/kg KG und Tag und der NOAEL für Fertilitätsbeeinträchtigung bei 150 mg EDAO/kg KG und Tag, der höchsten Dosis (The Dow Chemical Company 2008 b).

##### Hydrolyseprodukt 2-Amino-2-ethyl-1,3-propandiol

In der bereits erwähnten kombinierten Studie nach OECD-Prüfrichtlinie 422 auf Toxizität mit Screening-Test auf Reproduktions-/Entwicklungstoxizität mittels Schlundsondengabe an Sprague-Dawley-Ratten traten nach wiederholter Verabreichung bis zur höchsten Dosis von 1000 mg 2-Amino-2-ethyl-1,3-propandiol/kg KG keine substanzbedingten Effekte an den männlichen und weiblichen Reproduktionsorganen sowie auf den Östruszyklus auf (siehe Abschnitt 5.2.2.1; Bozo Research Center Co 2004).

In der bereits beschriebenen Studie nach OECD-Prüfrichtlinie 408 mit Schlundsondengabe an Wistar-Han-Ratten wiesen die männlichen und weiblichen Reproduktionsorgane bei der untersuchten höchsten Dosis von 1000 mg 2-Amino-2-ethyl-1,3-propandiol/kg KG und Tag keine substanzbedingten Befunde auf. Ebenso ergab die Untersuchung des Östruszyklus keine auffälligen Befunde (siehe Abschnitt 5.2.2.1; Charles River Laboratories 2024 a).

#### 5.5.2 Entwicklungstoxizität

In einer pränatalen Entwicklungstoxizitätsstudie ähnlich der OECD-Prüfrichtlinie 414 (Abweichung: keine Aufzeichnung des Futterverbrauchs) wurden je 25 trächtige Crl:CD^®^ BR-Sprague-Dawley-Ratten mit 0, 50, 250 oder 650 mg EDAO/kg KG und Tag (Reinheit: mindestens 93 %, Greim 1993) per Schlundsonde (Vehikel: entionisiertes Wasser, 10 ml/kg KG) vom 6. bis zum 15. Gestationstag behandelt. Die Auswahl der Dosierungen beruhte auf einer vorangegangenen Dosisfindungsstudie (k. w. A.). Die Untersuchung der Muttertiere erfolgte am 20. Gestationstag. Bei 650 mg/kg KG und Tag war die Körpergewichtszunahme der Muttertiere während der Behandlungsperiode sowie bezogen auf die gesamte Gestation verringert. Bei der höchsten Dosis von 650 mg EDAO/kg KG und Tag kam es bei 18/352 Feten (5,1 %) bzw. 4/24 Würfen (16,7 %) zu externen Fehlbildungen sowie bei 4/181 Feten (2,2 %) bzw. 2/24 Würfen (8,3 %) zu viszeralen Fehlbildungen. Dabei handelte es sich um seltene und ungewöhnliche Fehlbildungen. In der Kontrollgruppe lag die Inzidenz der externen Fehlbildungen bei 1/299 Feten (0,3 %) bzw. 1/20 Würfen (5,0 %) und die der viszeralen Fehlbildungen bei 1/150 Feten (0,01 %) bzw. 1/20 Würfen (5,0 %). Beispielsweise für Gaumenspalten lag die Inzidenz in der höchsten Dosisgruppe bei 14/352 Feten (0,28 %) bzw. 3/24 Würfen (12,5 %), bei den Kontrollen bei 0/299 Feten (0 %) bzw. 0/20 Würfen (0 %) und bei den historischen Kontrollen (k. A. zum Zeitraum) bei 3/20261 Feten (0,01 %) bzw. 3/1345 Würfen (0,22 %). Die Mehrheit der Fehlbildungen waren externe Fehlbildungen wie Gaumenspalten und Defekte des Verschlusses der ventralen abdominalen und/oder thorakalen Mittellinie (Nabelschnurhernie im Bereich des Darms, Omphalozele und Thorako-Gastroschisis). Diese kamen vorwiegend in zwei Würfen vor (4/4 lebende Feten bzw. 11/13 lebende Feten). In diesen beiden Würfen hatten die Feten die geringsten Körpergewichte und vermehrt verzögerte Ossifikationen der Sternebrae. Auch 2/3 Spätresorptionen eines Wurfes der höchsten Dosisgruppe wiesen ähnliche externe Fehlbildungen auf. Das Körpergewicht der Feten pro Wurf war bei 650 mg EDAO/kg KG und Tag erniedrigt (jedoch nicht statistisch signifikant, 3,4 ± 0,31 mg, Kontrolle: 3,6 ± 0,23). Bei 50 und 650 mg EDAO/kg KG und Tag war die Inzidenz an verzögerten Ossifikationen der Sternebrae erhöht (nicht statistisch signifikant). Zusammen mit den erniedrigten Fetengewichten und den bei 650 mg/kg KG und Tag aufgetretenen Fehlbildungen wurden die Ossifikationsverzögerungen bei dieser Dosis als Störungen der fetalen Entwicklung angesehen. Bei der höchsten Dosis kam es auch zu drei Spätresorptionen, die einen Wurf betrafen; in den anderen Dosisgruppen und der Kontrollgruppe hingegen wurden keine Spätresorptionen beobachtet. Die Entwicklungstoxizität war charakterisiert durch ausgeprägte Unterschiede zwischen einzelnen Würfen. Die Anzahl der Corpora lutea, der Implantationsstellen, der Postimplantationsverluste, der lebenden Feten sowie das Geschlechterverhältnis blieben durch die Behandlung unbeeinflusst. Der NOAEL für Entwicklungs- und Maternaltoxizität liegt bei 250 mg/kg KG und Tag (WIL Research Laboratories 1989). Aus der Studie ist nicht zu entnehmen, ob die gleichen Vatertiere für die beiden auffälligen Würfe eingesetzt wurden.

In der bereits beschriebenen 2-Generationenstudie an Ratten kam es bis zur höchsten Dosis von 150 mg EDAO/kg KG und Tag bis zum 4. Postnataltag nicht zu Effekten auf die Nachkommen (Abschnitt 5.5.1; The Dow Chemical Company 2008 b). Daraus leitet sich ein NOAEL für Perinataltoxizität von 150 mg/kg KG und Tag ab.

##### Hydrolyseprodukt 2-Amino-2-ethyl-1,3-propandiol

In einer Studie zur pränatalen Entwicklungstoxizität nach OECD-Prüfrichtlinie 414 mit Schlundsondengabe wurde je 22 trächtigen Wistar-Han-Ratten pro Dosisgruppe 0, 100, 300 oder 1000 mg 2-Amino-2-ethyl-1,3-propandiol/kg KG und Tag (Reinheit 92,80 %) in ultrareinem Wasser verabreicht. Bis zur höchsten Dosis von 1000 mg 2-Amino-2-ethyl-1,3-propandiol/kg KG und Tag kam es weder zu substanzbedingten maternal- noch zu entwicklungstoxischen Effekten einschließlich solche auf die Schilddrüsenhormone bei den Muttertieren und den anogenitalen Abstand bei den Feten. Damit liegt der NOAEL für Maternal- und Entwicklungstoxizität bei 1000 mg 2-Amino-2-ethyl-1,3-propandiol/kg KG und Tag, der höchsten Dosis (Charles River Laboratories 2024 b).

In der bereits erwähnten kombinierten Studie nach OECD-Prüfrichtlinie 422 nach wiederholter Verabreichung auf Toxizität mit Screening-Test auf Reproduktions-/Entwicklungstoxizität mittels Schlundsondengabe an Sprague-Dawley-Ratten kam es bis zur höchsten Dosis von 1000 mg 2-Amino-2-ethyl-1,3-propandiol/kg KG und Tag nicht zu substanzbedingten Effekten auf die Wurfparameter (siehe Abschnitt 5.2.2.1 und 5.5.1; Bozo Research Center Co 2004).

##### Hydrolyseprodukt Formaldehyd

Formaldehyd hat sich in pränatalen Entwicklungstoxizitätsstudien bei Ratten bis zur höchsten getesteten Konzentration von 40 ml/m^3^, bei Mäusen bis zur höchsten getesteten oralen Dosis von 185 mg/kg KG und Tag, bei Hunden bis zur höchsten getesteten oralen Dosis von 9,4 mg/kg KG und Tag und bei Goldhamstern (bis zur höchsten getesteten dermalen Konzentration einer Formaldehydlösung von 37 %) nicht als teratogen herausgestellt. Die niedrigste Effektkonzentration bei Ratten nach Inhalation lag bei 20 ml/m^3^, bei der erniedrigte Fetengewichte festgestellt wurden. Die NOAEC für Entwicklungstoxizität betrug 10 ml/m^3^ ohne Maternaltoxizität (Henschler 1991).

### 5.6 Genotoxizität

#### 5.6.1 In vitro

Die genauen Daten der In-vitro-Genotoxizitäts-Studien finden sich in Tabelle 5.

Bakterielle Mutagenitätstests mit den Salmonella-Stämmen TA98, TA100, TA1535, TA1537 und TA1538 zeigten mit und ohne metabolischem Aktivierungssystem keinen relevanten Anstieg der Revertantenanzahl (Greim 1993).

Primäre Rattenhepatozyten wurden mit 0,1–100 µl EDAO/ml und 10 µCi/ml ^3^H-Thymidin 18–20 Stunden inkubiert. Es erfolgte keine veränderte DNA-Reparatursynthese in diesem UDS-Test (ECHA 2013; Toxicon Corporation 1991 b).

EDAO führte nach einstündiger Inkubation im Konzentrationsbereich 0,25–4,0 µl/ml nicht zu einer Erhöhung der DNA-Reparatursynthese im UDS-Test. Zytotoxizität trat bei 5,0 µl/ml auf (Greim 1993; Toxicon Corporation 1987).

Nach Inkubation von CHO-Zellen mit 0,5–5 µl EDAO/ml oder 0,0125–0,2 µl EDAO/ml erfolgte mit und ohne Zusatz metabolischer Aktivierung keine Zunahme an Chromosomenaberrationen. Zytotoxizität trat ab der niedrigsten eingesetzten Konzentration auf (Toxicon Corporation 1988, 1991 a).

Ein TK^+/–^-Test mit L5178Y-Mauslymphomzellen nach OECD-Prüfrichtlinie 476 verlief ab 34,9 µM EDAO ohne Aktivierung und ab 52,4 µM mit Zugabe eines metabolischen Aktivierungssystems positiv. Die getrennte Erfassung großer und kleiner Kolonien deutet darauf hin, dass aufgrund der deutlichen Zunahme kleiner Kolonien hauptsächlich Chromosomenaberrationen vorlagen. In Anwesenheit von Formaldehyd-Dehydrogenase und des Cofaktors Nikotinamidadenindinukleotid (NAD^+^) traten keine Effekte auf. Auch eine initial mutagene Konzentration an Formaldehyd führte in diesem modifizierten Test zu negativen Ergebnissen. Daraus kann geschlossen werden, dass die hydrolytische Abspaltung von Formaldehyd den genotoxischen Effekt bewirkte (Charles et al. 2005).

Ein weiterer TK^+/–^-Test nach OECD-Prüfrichtlinie 476 führte bei L5178Y-Mauslymphomzellen bei Konzentrationen von 1,25–10 µg/ml ohne metabolische Aktivierung ab 5 µg/ml und in einem Wiederholungsversuch ab 6 µg/ml zu häufigeren Mutationen. Als Maß für Zellviabilität betrug das „relative total growth“ (RTG) 67–10 % bzw. 82–21 %. Bei Zugabe eines metabolischen Aktivierungssystems konnte bei Konzentrationen von 1,25–24 µg/ml eine Zunahme an Mutanten ab 7,5 µg/ml und im Wiederholungsversuch ab 10 µg/ml beobachtet werden. Das RTG betrug 86–16 % bzw. 110–9 %. Es trat eine deutliche Zunahme kleiner Kolonien auf, das deutet auf Chromosomenaberrationen hin (ECHA 2013; The Dow Chemical Company 2002 a).

**Tab. 5:** In-vitro-Genotoxizität von EDAO

**Endpunkt (Testmethode)**	**Testsystem**	**Konzentrationen**	**wirksame Konzentration^a)^**	**Zytotoxizität^a)^**	**Ergebnis^a)^**		**Literatur**
					**–m. A.**	**+m. A.**	
Genmutation	S. typhimurium TA98, TA100, TA1535, TA1537, TA1538	–m. A.: 0–600 µg/Platte, +m. A.: 0–600 µg/Platte	–	–	–	–	Greim 1993
UDS	Rattenhepatozyten	0; 0,5; 1,0; 5,0; 10; 50; 100 µl/ml	–	> 100 µl/ml	–	n. u.	ECHA 2013; Toxicon Corporation 1991 b
	Rattenhepatozyten	0; 0,25–5,0 µl/ml	–	5 µl/ml	–	n. u.	Greim 1993; Toxicon Corporation 1987
CA	CHO-Zellen	0; 0,5; 1,0; 2,0; 3,0; 4,0; 5,0 µl/ml	–	5,0 µl/ml	–	–	Toxicon Corporation 1988
	CHO-Zellen	0; 0,0125; 0,025; 0,05; 0,1; 0,2 µl/ml	–	–	–	–	ECHA 2013; Toxicon Corporation 1991 a
Genmutation (Tk^+/–^)	Mauslymphomzellen L5178Y	0; 8,7; 17,5; 34,9; 52,4; 69,8; 90,9; 104,7; 136,4 µM	–m. A.: 34,9 µM, +m. A.: 52,4 µM	104,7 µM	+	+	Charles et al. 2005
	Mauslymphomzellen L5178Y, unter Zusatz von FDH (0,1 U) und NAD^+^ (8 mM)	0; 55,9; 69,8 µM	–m. A.: 34,9 µM, +m. A.: 52,4 µM	104,7 µM	–	n. u.	Charles et al. 2005
	Mauslymphomzellen L5178Y	0; 1,25–100 µg/ml	–m. A.: 5 µg/ml, +m. A.: 7,5 µg/ml	15 µg/ml	+	+	ECHA 2013; The Dow Chemical Company 2002 a
	Mauslymphomzellen L5178Y	–m. A.: 0; 0,5–16 µg/ml, +m. A.: 0; 2–24 µg/ml	–m. A.: 8 µg/ml, +m. A.: 14 µg/ml	12 µg/ml	+	+	ECHA 2013

^a)^ Ergebnis statistisch signifikant, wenn nicht anders angegeben

–: negatives Ergebnis; +: positives Ergebnis; CA: Chromosomenaberrationen; FDH: Formaldehyd-Dehydrogenase; –/+m. A.: ohne/mit metabolischer Aktivierung; NAD^+^: Nikotinamidadenindinukleotid; n. u.: nicht untersucht; UDS: DNA-Reparatursynthese

##### Hydrolyseprodukt 2-Amino-2-ethyl-1,3-propandiol

In vitro zeigte 2-Amino-2-ethyl-1,3-propandiol keine Genotoxizität in bakteriellen Mutagenitätstests, in einem Chromosomenaberrationstest und einem HPRT-Genmutationstest mit CHO-Zellen (ECHA 2018).

#### 5.6.2 In vivo

In einem UDS-Test nach OECD-Prüfrichtlinie 486 wurden nach einmaliger Schlundsondengabe von 0, 1000 oder 2000 mg/kg KG an vier F344-Ratten 2–4 oder 14–16 Stunden später Primärkulturen der Hepatozyten mit 10 µCi ^3^H-Thymidin/ml (35–60 Ci/mmol) vier Stunden inkubiert. Die DNA-Synthese war im Vergleich zur Kontrolle nicht verändert (Charles et al. 2005; Covance Laboratories, Inc. 2002).

Je fünf bis sechs männliche CD-1-Mäuse erhielten an zwei aufeinander folgenden Tagen per Schlundsonde 0, 500, 1000 oder 2000 mg/kg KG. Die 24 Stunden nach der letzten Gabe durchgeführte Untersuchung ergab keine Erhöhung an Mikronuklei in polychromatischen Erythrozyten im Knochenmark und auch der Anteil an polychromatischen Erythrozyten als Maß für Knochenmarkstoxizität war nicht statistisch signifikant verändert. Bei der höchsten Dosis starben drei der acht behandelten Mäuse. Der Test wurde nach OECD-Prüfrichtlinie 474 durchgeführt (Charles et al. 2005; The Dow Chemical Company 2002 b).

##### Hydrolyseprodukt 2-Amino-2-ethyl-1,3-propandiol

Hierzu liegen keine Daten vor.

### 5.7 Kanzerogenität

Hierzu liegen keine Daten vor.

## 6 Bewertung

Kritische Effekte sind die Reizwirkung an der Haut und die ätzende Wirkung am Auge. Beim Hydrolyseprodukt Formaldehyd sind die kanzerogene Wirkung an der Nase sowie die hautsensibilisierende Wirkung die empfindlichsten Endpunkte. Beim 2-Amino-2-ethyl-1,3-propandiol als weiterem Hydrolyseprodukt steht ebenfalls die Reizwirkung im Vordergrund.

**Krebserzeugende Wirkung. **Die lokale Kanzerogenität des Hydrolyseprodukts Formaldehyd ist ausführlich dokumentiert (Greim 2000). Formaldehyd ist in Kanzerogenitäts-Kategorie 4 eingestuft, da es bei Konzentrationen, die die Entgiftungskapazitäten des Nasengewebes überschreiten, dort kanzerogen wirkt.

Untersuchungen zur krebserzeugenden Wirkung mit EDAO liegen nicht vor. Die Genotoxizität am wahrscheinlichen Zielorgan Nase aufgrund der Formaldehyd-Freisetzung ist nicht zu beurteilen, da Inhalationsstudien fehlen.

Aufgrund der hydrolytischen Freisetzung von Formaldehyd im Nasengewebe ist die kanzerogene Wirkung von Formaldehyd auch bei Exposition gegen EDAO zu erwarten.

In Analogie zu Formaldehyd wird EDAO in die Kanzerogenitäts-Kategorie 4 eingestuft. Bei Einhaltung des MAK-Werts von EDAO ist kein Beitrag zum Krebsrisiko beim Menschen zu erwarten.

**MAK-Wert. **EDAO ist hautreizend und führt zu schweren Augenschäden.

Formaldehyd zeigt deutliche Reizwirkungen in der Nase und kann bei chronischer Exposition Tumoren im Epithel der Nasenschleimhaut erzeugen. Die Entgiftungskapazitäten des Nasengewebes für Formaldehyd werden bei einem MAK-Wert von 0,3 ml/m^3^ nicht überschritten (Greim 2000; Hartwig 2010).

2-Amino-2-ethyl-1,3-propandiol wirkt stark reizend am Auge, was auf die hohe Basizität (pH-Wert 10,8 bei 0,1 M; pKs-Wert 9,03) zurückgeführt werden kann (ECHA 2018; IFA 2023 a).

Bei pH 7,0 und 25 °C hat EDAO in wässriger 0,002%iger Lösung eine Halbwertszeit von 1,9 Stunden, d. h., dass EDAO nur mäßig schnell hydrolysiert. Der berechnete und der gemessene Dampfdruck bei 25 °C liegen mit 1,04 hPa bzw. 0,606 hPa in dem Grenzbereich, ab dem zusätzlich zum Dampf die Aerosolfraktion mitbestimmt werden sollte (DFG 2024). Es kann jedoch davon ausgegangen werden, dass bei niedrigen Konzentrationen bei diesem Dampfdruck keine Aerosole auftreten.

Ausgehend von dem Worst-Case einer vollständigen Freisetzung von zwei Molekülen Formaldehyd kann in Analogie zum MAK-Wert von 0,3 ml/m^3^ für Formaldehyd für EDAO ein MAK-Wert von 0,15 ml/m^3^ abgeleitet werden. Aufgrund der Halbwertszeit von 1,9 Stunden bei 25 °C kann davon ausgegangen werden, dass keine sofortige vollständige Freisetzung von Formaldehyd an der Nasenschleimhaut im oberen Atemtrakt stattfindet. Die Entgiftung von Formaldehyd durch mukoziliäre Clearance sowie durch Inaktivierung in den Epithelzellen erfolgt also parallel mit der hydrolytischen Entstehung. Dies bedeutet, dass hier ein Sicherheitsabstand vorliegt.

Eine zusätzliche Wirkung des Hydrolyseproduktes 2-Amino-2-ethyl-1,3-propandiol muss mitbetrachtet werden. 2-Amino-2-ethyl-1,3-propandiol zeigt ebenso wie EDAO eine Reizwirkung, die jedoch im direkten Vergleich der Studien zur Reizwirkung an Haut und Auge schwächer als beim EDAO ausfällt (Abschnitt 5.3). Für 2-Amino-2-ethyl-1,3-propandiol liegt kein MAK-Wert vor. Im Vergleich mit einem weiteren Amin, dem 2-Aminoethanol mit einem MAK-Wert von 0,2 ml/m^3^, das stärker basisch ist und einen höheren pKs-Wert (Laube et al. 2016) als 2-Amino-2-ethyl-1,3-propandiol aufweist, kann eine geringere Reizwirkung des 2-Amino-2-ethyl-1,3-propandiols im oberen Atemtrakt abgeschätzt werden. Das Konzept der Dosis-Addition bei zwei reizenden Substanzen wird hier nicht angewendet, da die Wirkungsmechanismen von Formaldehyd und 2-Amino-2-ethyl-1,3-propandiol nicht gleich sind und die Wirkstärke des Formaldehyds höher ist. Es kann also davon ausgegangen werden, dass auch bei vollständiger Hydrolyse bei einer Konzentration von 0,15 ml EDAO/m^3^ keine Wirkungsverstärkung des freigesetzten Formaldehyds durch die zusätzliche Reizwirkung von 2-Amino-2-ethyl-1,3-propandiol auftritt.

Daher wird ein MAK-Wert von 0,15 ml/m^3^ bzw. 0,89 mg/m^3^ für EDAO festgelegt.

**Hinweis für Anwendung im Kühlschmierstoff: **Bei Anwendung in verdünnten wässrigen Lösungen sollte mit einer vollständigen Hydrolyse gerechnet und daher der MAK-Wert für Formaldehyd (Greim 2000; Hartwig 2010) eingehalten werden.

**Spitzenbegrenzung. **In Analogie zu Formaldehyd wird der Stoff in Spitzenbegrenzungs-Kategorie I eingestuft und ein Überschreitungsfaktor von 2 festgesetzt.

**Fruchtschädigende Wirkung. **In einer pränatalen Entwicklungstoxizitätsstudie, zum Großteil durchgeführt nach OECD-Prüfrichtlinie 414, an Crl:CD^®^ BR-Sprague-Dawley-Ratten mit Schlundsondengabe vom 6. bis zum 15. Gestationstag, traten bei der höchsten Dosis von 650 mg EDAO/kg KG und Tag vor allem externe Fehlbildungen wie Gaumenspalten und Defekte des Verschlusses der ventralen abdominalen und/oder thorakalen Mittellinie sowie Spätresorptionen und verzögerte Ossifikationen der Sternebrae auf. Dabei zeigten sich ausgeprägte Unterschiede zwischen einzelnen Würfen. Bei der höchsten Dosis war die Körpergewichtszunahme der Muttertiere verringert. Der NOAEL für Entwicklungs- und Maternaltoxizität lag bei 250 mg EDAO/kg KG und Tag (Greim 1996; WIL Research Laboratories 1989).

Aus einer Studie nach OECD-Prüfrichtlinie 416 an Crl:CD(SD)-Ratten mit Schlundsondengabe leitet sich ein NOAEL für Perinataltoxizität von 150 mg/kg KG und Tag, der höchsten Dosis, ab (The Dow Chemical Company 2008 b).

Zur toxikokinetischen Übertragung dieser NOAEL in eine Konzentration in der Luft am Arbeitsplatz werden berücksichtigt: die tägliche Exposition der Tiere im Vergleich zur fünftägigen Exposition pro Woche am Arbeitsplatz (7:5, nur für die 2-Generationenstudie), der dem toxikokinetischen Unterschied zwischen der Ratte und dem Menschen entsprechende speziesspezifische Korrekturwert (1:4), die orale Resorption von 100 % (experimentelle Bestimmung), das Körpergewicht (70 kg) und das Atemvolumen (10 m^3^) des Menschen sowie die angenommene 100%ige inhalative Resorption. Damit errechnen sich entsprechende Konzentrationen von 438 mg/m^3^ bzw. 368 mg/m^3^.

Da bei Ratten nur bei maternaltoxischen Dosierungen Ossifikationsverzögerungen, Fehlbildungen und Spätresorptionen auftreten, der Unterschied zwischen einzelnen Würfen für die entwicklungstoxischen Effekte sehr groß ist, und der 492- bzw. 413-fache Abstand der NOAEC für Entwicklungstoxizität zum MAK-Wert von 0,89 mg/m^3^ ausreichend groß ist, wird EDAO der Schwangerschaftsgruppe C zugeordnet.

**Keimzellmutagene Wirkung. **EDAO war nicht genotoxisch in einem UDS-Test, nicht mutagen in Bakterien und nicht klastogen in einem Chromosomenaberrationstest. In mehreren TK^+/–^-Tests wurde jedoch eine genotoxische Wirkung festgestellt, die hauptsächlich durch Chromosomenaberrationen bedingt ist und auf Formaldehyd zurückgeführt wurde. In-vivo-Tests auf Mikronuklei und DNA-Schäden verliefen negativ. Untersuchungen an Keimzellen fehlen.

In vitro war 2-Amino-2-ethyl-1,3-propandiol nicht genotoxisch.

Das Hydrolyseprodukt Formaldehyd ist in Kategorie 5 für Keimzellmutagene eingestuft. Dies bedeutet, dass durch Inhalation aufgenommenes Formaldehyd unter Einhaltung des MAK-Wertes von 0,3 ml/m^3^ einen sehr geringen Beitrag zum genetischen Risiko für den Menschen erwarten lässt (Greim 2000). Dies gilt in Analogie auch für EDAO und die Substanz wird daher in Kategorie 5 eingestuft.

**Hautresorption. **Bei einer dermalen Resorptionsstudie nach OECD-Prüfrichtlinie 417, bei der Ratten beiderlei Geschlechts mit 5 mg radioaktiv-markierten EDAO/kg KG semiokklusiv für sechs Stunden exponiert wurden, betrug die dermale Resorption 27 ± 3 % und 25 ± 5 %. Aus der Studie ergibt sich ein Flux von 4,5 µg/cm^2^ und Stunde, bei 2000 cm^2^ und einer Stunde also 9 mg aufgenommenes EDAO. Die Resorption erfolgt schnell, was durch maximale Plasmakonzentrationen 2–3 Stunden nach Applikation dokumentiert wurde.

Der systemische NOAEL beträgt 15 mg/kg KG in einer oralen 90-Tage-Studie an Hunden. Umgerechnet in eine Konzentration in der Luft wären das 8,75 mg/m^3^ (15 mg/kg KG/1,4 (toxikokinetischer Umrechnungsfaktor Hund) × 70 kg/10 m^3^/6 (Zeitextrapolation, weil eine 90-Tage-Studie beim Hund sehr kurz ist im Vergleich zur Lebensdauer)/2 (Übertragung Tierversuchsdaten auf Mensch) × 7/5 (7-Tage-Applikation) × 1 (orale Resorption)). Bei 100 % inhalativer Resorption und 10 m^3^ Atemvolumen ergibt sich eine tägliche tolerable Aufnahme von 88 mg. Aus der oralen 90-Tage-Studie an Ratten mit dem systemischen NOAEL von 50 mg/kg KG errechnet sich eine Konzentration von 30,6 mg/m^3^ (50 mg/kg KG/4 (toxikokinetischer Umrechnungsfaktor Ratte) × 7/5 (7-Tage-Applikation) × 70 kg/10 m^3^/2 (Zeitextrapolation)/2 (Übertragung Tierversuchsdaten auf Mensch) × 1 (orale Resorption)). Bei 100 % inhalativer Resorption und 10 m^3^ Atemvolumen ergibt sich eine tägliche tolerable Aufnahme von 306 mg.

Unter der Annahme einer raschen Hydrolyse des EDAO mit Bildung von Formaldehyd lässt sich die Zunahme des Formaldehydspiegels im Blut nach dermaler Applikation abschätzen: aus den zuvor berechneten insgesamt 9 mg EDAO (0,06 mmol) werden 2 × 0,06 mmol Formaldehyd = 3,6 mg Formaldehyd innerhalb einer Stunde freigesetzt, pro Minute demnach etwa 60 µg bzw. 75 µg/1,25 Minuten (mittlere Halbwertszeit des Formaldehyds im Blut; Kaden et al. 2010).

Die transdermale Aufnahme von EDAO in den letzten sechs Halbwertszeitintervallen führt demnach zu einer im Blut zirkulierenden Formaldehydmenge von (75 + 38 + 19 + 9 + 5 + 2) µg = 0,15 mg.

Der physiologisch bedingte Formaldehydspiegel im Blut des Menschen beträgt etwa 2–3 mg/l (10–15 mg in 5 l Blut) (Heck et al. 1985). Der zusätzliche Eintrag durch transdermal aufgenommenes EDAO erhöht demnach die Gleichgewichtskonzentration von Formaldehyd im Blut nicht signifikant.

Da die über die Haut aufgenommene Menge weniger als 25 % der systemisch tolerablen Menge beträgt und die Freisetzung von Formaldehyd nicht zu einer bedeutsamen Erhöhung des Formaldehydspiegels im Blut führt, wird EDAO nicht mit „H“ markiert.

**Sensibilisierende Wirkung. **Den klinischen Beobachtungen beim Menschen zufolge weist EDAO ein kontaktallergenes Potenzial auf und stellt ein beruflich relevantes Kontaktallergen für die Gruppe der Metallarbeiter dar. Die vorliegenden Ergebnisse in tierexperimentellen Untersuchungen sind uneinheitlich, lassen aber ebenfalls eine kontaktsensibilisierende Wirkung erkennen. Insgesamt erfolgt auch aufgrund der Eigenschaft der Substanz, Formaldehyd freizusetzen, weiterhin eine Markierung mit „Sh“. Daten zur sensibilisierenden Wirkung an den Atemwegen fehlen, sodass weiterhin keine Markierung mit „Sa“ erfolgt.
